# Development of a high-grade glioma preclinical surgery model using an inducible KRAS/TP53 Oncopig

**DOI:** 10.3389/fonc.2026.1810135

**Published:** 2026-04-20

**Authors:** Caleb Y. Kwon, Ryan B. Duke, Augustino V. Scorzo, Kirk Maurer, Linton Evans, Rendall R. Strawbridge, William R. Warner, Chengpei Li, Xiaoyao Fan, Allison Tomasino, Ciara Groesbeck, George Zanazzi, Caitlin Evers, P. Jack Hoopes, David W. Roberts, Scott C. Davis, Keith D. Paulsen

**Affiliations:** 1 Thayer School of Engineering, Dartmouth College, Hanover, NH, United States; 2 Center for Comparative Medicine and Research, Dartmouth College, Lebanon, NH, United States; 3 Dartmouth Cancer Center, Lebanon, NH, United States; 4 Section of Neurosurgery, Dartmouth-Hitchcock Medical Center, Lebanon, NH, United States; 5 Geisel School of Medicine, Dartmouth College, Hanover, NH, United States; 6 Department of Pathology, Dartmouth-Hitchcock Medical Center, Lebanon, NH, United States

**Keywords:** AAV, brain, glioma, Oncopig, porcine, tumor

## Abstract

**Introduction:**

Surgery remains the primary treatment modality for high-grade glioma; yet, Q10 median overall survival has seen only modest improvement over the past few decades. Lack of reliable preclinical models that recapitulate the volumes and infiltrative nature of human tumors is a persistent challenge for development and evaluation of new surgical techniques. In this context, the Oncopig model has been shown to produce realistic tumors in other organs. It is a transgenic model that encodes for the oncogenic P53 and KRAS mutations which can be switched on through delivery of Cre-recombinase, usually using a viral vector, driving endogenous tumor induction. The objective of this study was to explore the potential of Oncopigs as preclinical high-grade glioma model.

**Methods:**

A cocktail of adeno-associated viruses (AAV) encoding Cre-recombinase was implanted in the brains of ten Oncopigs. Animals were then monitored for tumor growth using MRI. Once tumors were detected, an additional MRI was acquired several days later to track growth and animals then either underwent surgical resection or tissue harvest. Tumor specimens were collected for histopathological analysis.

**Results:**

Tumors were observed on MRI scans for six of the ten pigs. The median time to observed tumor onset was 24 days and ranged from 13 to 104 days. Tumor volumes prior to euthanasia in these animals ranged from 250 to 2000 mm3. Pathological analysis of tumor specimens revealed infiltrative tumors in six subjects, with features consistent with high-grade glioma. Fluorescence-guided surgical resection with ALA-PpIX in one animal revealed high levels of PpIX fluorescence, also consistent with high-grade gliomas in humans.

**Discussion:**

These results indicate that the Oncopig can be used to induce brain tumors that exhibit pathological features consistent with high-grade glioma in humans. A realistic high-grade glioma model such as this should support better preclinical development and evaluation of new surgical and other treatment strategies.

## 1 Introduction

High-grade glioma (HGG), encompassing WHO Grade III and IV tumors, represent the most common and aggressive primary cancers of the central nervous system in adults (1, 2). The current standard of care includes maximal safe surgical resection followed by concurrent and adjuvant radiation and chemotherapy (3–6). Despite this intense multimodal therapy, the median overall survival remains 14–16 months with little improvement seen in recent decades (5, 7). Given the uniformly poor prognosis, need exists for more effective therapeutic strategies.

In this context, animal models have been and continue to be essential in the development of new treatments including surgical interventions. To date, most preclinical systems employ small animals that fail to recapitulate certain elements of human gliomas or brain tumors, including the anatomical and physiological environment encountered at time of surgery (8–10). Size, scale and other anatomical differences associated orthotopic small animal brain tumor models limit evaluation of surgical technologies in translational studies (11, 12). To overcome these barriers, a porcine xenograft model was recently introduced to establish a preclinical *in vivo* system that more closely mimics human anatomy for surgical guidance research (13–16). This model relies on chronic immunosuppression of a wild type minipig followed by stereotactic implantation of a commonly used human glioma cell line in the cerebral hemispheres. While the approach is more clinically relevant in complexity and size, the resulting tumors are well demarcated with sharp boundaries between tumor and normal tissue, features that are not characteristic of gliomas with diffuse infiltration. The required chronic immunosuppression represents a further deviation from the clinical environment encountered in humans which could affect study results and their translational relevance. The introduction of a large animal model that harbors HGG features and diffuse, infiltrative borders would be a major advance for translational research of new surgical technologies.

Recently, a transgenic “Oncopig” model system was introduced to facilitate use of more anatomically and biologically relevant tumors (17–21). These animals were genetically engineered to harbor transgenes in their genome for the hallmark oncogenic mutations KRAS^G12D^ and TP53^R167H^. These transgenes are positioned next to a STOP cassette within a Cre-lox system such that they are not expressed until the cassette is removed by the introduction of Cre-recombinase enzymes. Removal of the cassette then permits expression of the mutations, resulting in organ-specific, spontaneous initiation of tumors at the site of Cre-recombinase administration. To introduce the enzyme into cells, viral vectors containing a plasmid encoding for Cre-recombinase are administered locally. Prior efforts using similar models delivered viral vectors to induce tumors successfully in several organs (22–29). We also demonstrated this in a previous publication on the induction of a tumor in a single Oncopig (21). However, to our knowledge, a comprehensive study of glioma induction with this model system has not been reported.

Herein, we report on development and characterization of a porcine inducible high-grade glioma model using the Oncopig platform in multiple animals. By locally delivering a cocktail of adeno-associated viruses (AAVs) encoding Cre-recombinase into the brain, we induced the formation of brain tumors in these animals. After tumor growth was confirmed via contrast-enhanced magnetic resonance imaging (CE-MRI), tumor and healthy brain tissue samples were collected for pathological tumor characterization. The model was also used for evaluating new technologies for neurosurgical image and fluorescence guidance described elsewhere (21).

## 2 Methods

### 2.1 Oncopig model

All procedures were conducted in accordance with protocols approved by the Institutional Animal Care and Use Committee at Dartmouth College. The Oncopigs (OPs, N = 10) in this study were purchased from the National Swine and Resource and Research Center (NSRRC:0033, Columbia, Missouri, United States). Original development of the OP model was described previously by Schachtschneider, et al. (19). Briefly, OP is a transgenic swine model which utilizes a Cre-Lox system that prevents downstream expression of KRAS^G12D^ and TP53^R167H^ driver mutations. Upon introduction of the enzyme Cre-recombinase, the loxP sites are excised, allowing for expression of driver mutations leading to tumorigenesis. Cre-recombinase has typically been introduced through local administration of adenovirus carrying a Cre-recombinase plasmid payload. In this study, we administered a cocktail of adeno-associated viral vectors in the brain to deliver the Cre-recombinase plasmid.

Once animals were received, they were acclimated for at least 2 weeks before the first imaging session. Animals were sedated for MRI scanning, intracranial inoculation and tumor resection procedures. Sedation involved administering Ketamine (20 mg/kg IM) and Midazolam (0.4 mg/kg IM) and intubating the animals for anesthesia maintenance using Isoflurane (1-4%, 95% oxygen). An IV catheter was secured in an ear vein for fluid management and body temperature was maintained at 37.5° C. Heart rate, respiration rate, end-tidal CO_2_, and SpO_2_ levels were monitored continuously throughout the surgical procedure.

### 2.2 Viral vectors for delivery of Cre-recombinase

With the exception of one animal (OP1), all were administered a cocktail consisting of equal parts of the following adeno-associated viral vectors: *pENN.AAV5.hSyn.Cre.WPRE.hGH* (Addgene105535-AAV5, Watertown, Massachusetts, United States), *pENN.AAV2.CMVs.PI.Cre.rBG* (Addgene 105537-AAV2), *pENN.AAV9.CMVs.PI.Cre.rBG* (Addgene 105537-AAV9), and *AAV5.GFAP(2.2).iCre* (Vector Biolabs VB1172, Malvern, Pennsylvania, United States).

This cocktail was prepared by combining 4 µL of each viral vector into 20 µL aliquots. Each aliquot was then stored at -80 °C until the time of implantation. OP1 received only *AAV5.GFAP(2.2).iCre*. In either case, the concentration of the solution was 10^13^ vg/mL.

### 2.3 Intracranial inoculation of viral vectors

**Figure 1:**
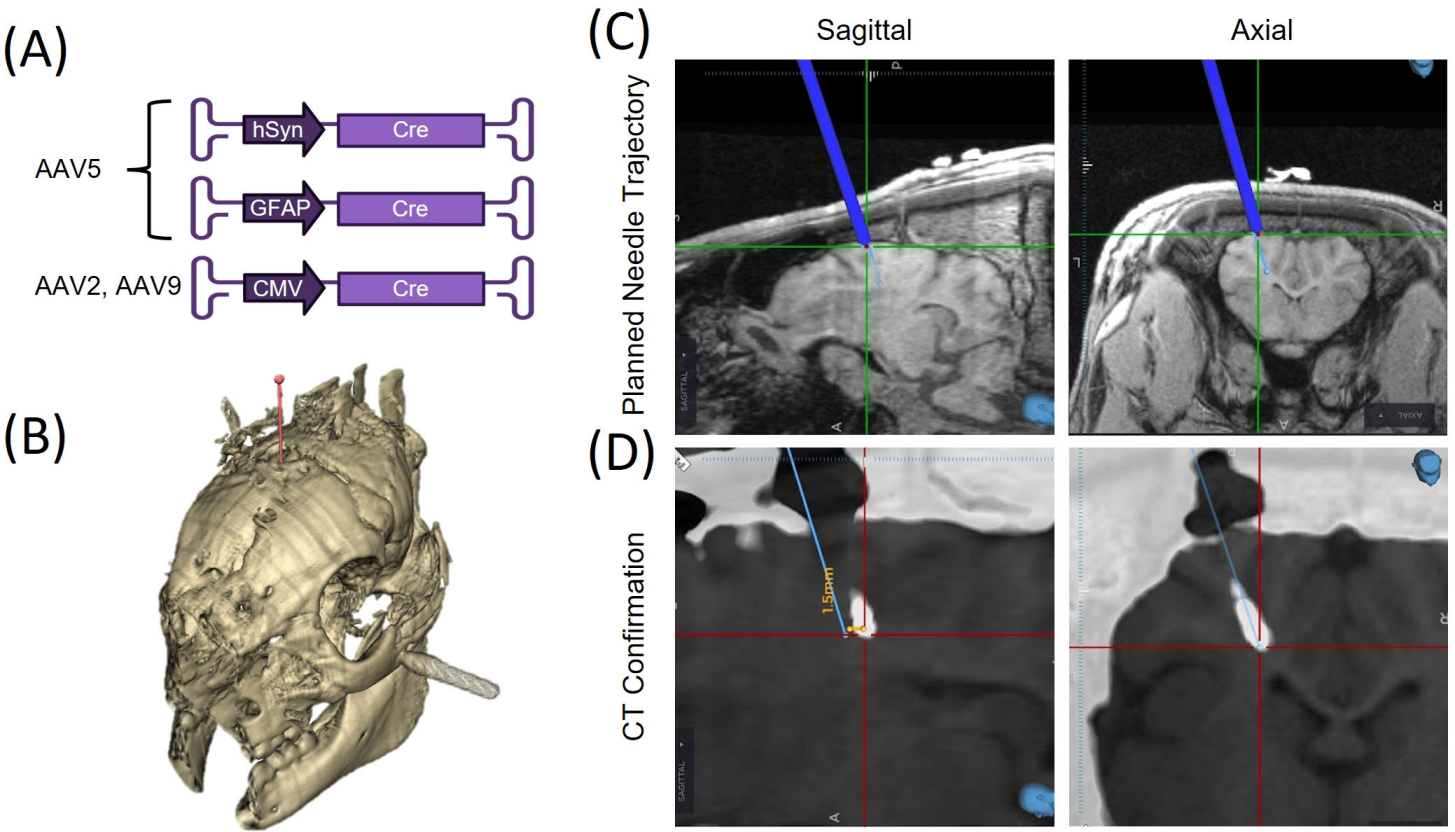
**(A)** Adeno-associated virus (AAV) illustration composed of a mixture of AAV serotypes (2, 5, and 9) with distinct promoters (hSyn, CMV, GFAP) to drive Cre-recombinase expression and initiate tumor growth. **(B)** Skull rendering from a CT scan of one of the animals with the planned needle trajectory for AAV-implantation. **(C)** Sagittal and axial views of pre-operative T1-weighted MRI showing the planned needle trajectory in blue. **(D)** CT images acquired during needle insertion superimposed on co-registered MR images to confirm implantation location.

All animals were anesthetized for the intracranial inoculation procedure as described previously. Much of the procedure was consistent for all animals (**Figure 1**), although some differences occurred in imaging use and injection processes, as described below.

OP1 did not undergo pre-procedure imaging. The animal was positioned in a Kopf 1530 stereotactic frame (Kopf Instruments, Tujunga, CA). A 2 cm scalp incision was made 4 mm left of the midline to expose the skull. A 2 mm burr hole was then drilled at stereotactic coordinates 1 cm caudal to bregma and 4 mm left of the midline. A syringe (Hamilton, Franklin, MA) containing 20 µL *AAV5.GFAP(2.2).iCre* (Vector Biolabs VB1172, Malvern, Pennsylvania, United States), was advanced 1 cm deep to the skull surface. A total volume of 10 µL was injected in multiple small boluses over 8.5 minutes. A CT scan was acquired prior to needle withdrawal to record placement.

Inoculations for OPs 2–6 were completed with intraoperative image guidance. Animals were positioned in a custom MRI-compatible stereotactic head frame and both CT and MRI scans were acquired. The animal and preoperative images were registered to a Medtronic StealthStation S8 image-guidance system (Dublin, Ireland) using self-adhesive fiducials placed on the animal before imaging. Additionally, a Medtronic SureTrak instrument was affixed to a microinjector pump (UMP3, World Precision Instruments) holding the Hamilton syringe containing the vector cocktail, which enabled real-time tracking of the needle on pre-operative images. Next, a ~3cm incision was made to expose the skull and a cranial window (<1 cm) was drilled through the skull at stereotactic coordinates 1 cm caudal to bregma and either 4 mm left (for OPs 2, 3 and 5) or right (for OPs 4 and 6) of the midline. The needle was advanced into the brain and intraoperative CT scans were acquired to verify needle position in the brain. The microinjector pump infused 10 uL of the four-vector cocktail into the brain over the course of 30 min. and the needle was left in place for another 15 minutes before withdrawal.

For OPs 7-10, pre-operative CT and MRI scans were acquired and used to plan needle trajectories based on anatomical landmarks, targeting white matter ~1 cm caudal to bregma and ~1 cm right of midline. For the inoculation, animals were positioned in a stereotactic frame, an incision was made and a cranial window drilled as described previously and the AAV cocktail (10 µL) was injected in the same manner as described for OPs 2-6.

For all animals, bone wax was used to fill the trephination defect and the skin was sutured. Animals were recovered in an isolation cage and returned to their communal housing once recovered. Dexamethasone (1 mg/kg, IV) was administered immediately post-op and carprofen (4 mg/kg) was given SQ prior to the first surgical incision and orally within 24 h after the procedure.

### 2.4 MRI monitoring and image analysis

After inoculation, animals were monitored for tumor onset and growth using MRI every 2–4 weeks. T1-weighted, T2-weighted, T2-FLAIR, and contrast-enhanced T1-weighted images (CE-MRI) were acquired during each monitoring session. Animals were anesthetized and monitored as described for surgery. Specifically, the scan sequence involved the following procedure:

1. Prior to contrast, T1-weighted (TR: 2300 ms, TE: 3.39 ms, TI: 1060 ms, resolution: 0.5859 mm x 0.5859 mm x 1 mm and slice number = 120), T2-weighted (TR: 11720 ms, TE: 108 ms, resolution: 0.7812 mm x 0.7812 mm x 1.3 mm and slice number = 62), and FLAIR (TR: 9000 ms, TE: 85 ms, TI: 2500, resolution: 1 mm x 1 mm x 1.3 mm and slice number = 62) images were acquired.
2. Animals were then injected with a gadolinium-based contrast agent (Dotarem; Guerbet, Princeton, NJ, USA, dosed at 0.1mmol/kg, molecular weight: 754 Da) for contrast enhanced T1-weighted MRI (TR: 2300 ms, TE: 3.39 ms, TI: 1060 ms, resolution: 0.5859 mm x 0.5859 mm x 1 mm and slice number = 120).

Once tumors were visible, imaging frequency was increased to every 3–5 days until the tumor reached a diameter of ~1 cm, at which point animals were either euthanized for tissue harvesting and analysis or prepared for surgical resection of the tumor.

To quantify tumor volumes, enhancing regions in the CE-MRI image volumes were manually segmented using 3DSlicer (30) and volumes were calculated based on the segmented regions. The whole-brain was also segmented for visualization purposes.

### 2.5 Histopathology

Suspected tumor specimens were collected from full brain tissue harvesting or during surgical resection of the tumor. A total of 35 samples were collected from all Oncopigs for histopathological analysis.

Tissue specimens were fixed and stained with hematoxylin and eosin (H&E). Additionally, select specimens were processed with the following immunohistochemical (IHC) stains:

1. Epidermal growth factor receptor (EGFR, Abcam, Cat# ab52894)
2. Oligodendrocyte transcription factor 2 (OLIG2, Thermo, Cat# PA5-85734)
3. Glial fibrillary acidic protein (GFAP, Agilent, Cat# Z0334)
4. KRAS G12D (Abcam, Cat# ab221163)
5. P53 (Leica, Cat# PA0057)

Details on IHC staining procedures are included in the supplemental text. All tissue specimens were assessed by a neuropathologist for nuclear atypia, mitosis, microvascular proliferation and necrosis (AMEN score) in addition to the presence of tumor cell infiltration (31, 32).

### 2.6 Tumor resection

The model was evaluated in surgical guidance applications, and as an example, data from OP 4 are illustrated. This animal underwent surgical resection of tumor with 5-Aminolevulinic Acid-induced Protoporphyrin IX (ALA-PpIX) fluorescence and intraoperative MRI (iMRI). Three hours prior to resection, a 20 mg/kg human-equivalent dose of 5-ALA dissolved in sterile water (Thermo Fisher Scientific, Waltham, Massachusetts, United States) was delivered via an indwelling catheter in the lateral auricular vein. The animal was then prepared for surgery in the Center for Surgical Innovation surgical suite at Dartmouth-Hitchcock Medical Center with iMRI. A contrast-enhanced MRI was acquired at multiple timepoints throughout the tumor resection: preoperative, following craniotomy and durotomy, following partial resection (< 50% of the tumor volume), following near complete resection, and after complete resection of the tumor with no visible fluorescence. Conventional white light and fluorescence images were also acquired at corresponding time-points using a clinical Zeiss Kinevo neurosurgical microscope (Zeiss, Germany). Animals were anesthetized and monitored as described previously.

## 3 Results

### 3.1 Tumor development

Animals were monitored for tumor onset using MRI at regular intervals. Once a tumor was observed, a follow-up scan was completed several days later. **Table 1** provides the age and weight at inoculation, time between inoculation and observed tumor onset, and tumor volume prior to euthanasia for the animals that grew tumors. Throughout the study, none of the animals exhibited clinical signs of distress due to the implantation procedure or tumor growth.

**Table 1:** Summary of experimental parameters for each pig.

Pig ID	Implant age (Days)	Implant hemisphere	Implant weight (kg)	Days before tumor observed	Tumor volume at end of study (mm^3^)
OP1	54	Left	11	104	256.12
OP2	117	Left	37.2	13	1961.06
OP3	187	Left	47	21	802.55
OP4	56	Right	12	56	381.87
OP5	69	Left	11	20	525.28
OP6	72	Right	15.2	27	672.57
OP7	56	Right	11.9	–	–
OP8	56	Right	10.3	–	–
OP9	89	Right	27	–	–
OP10	89	Right	28.7	–	–

Tumors were observed in six of the ten subjects and the median time to observed tumor onset was 24 days and ranged from 13 to 104 days. Four animals were monitored for a minimum of 100 days but did not exhibit tumors on serial MRI.

**Figure 2:**
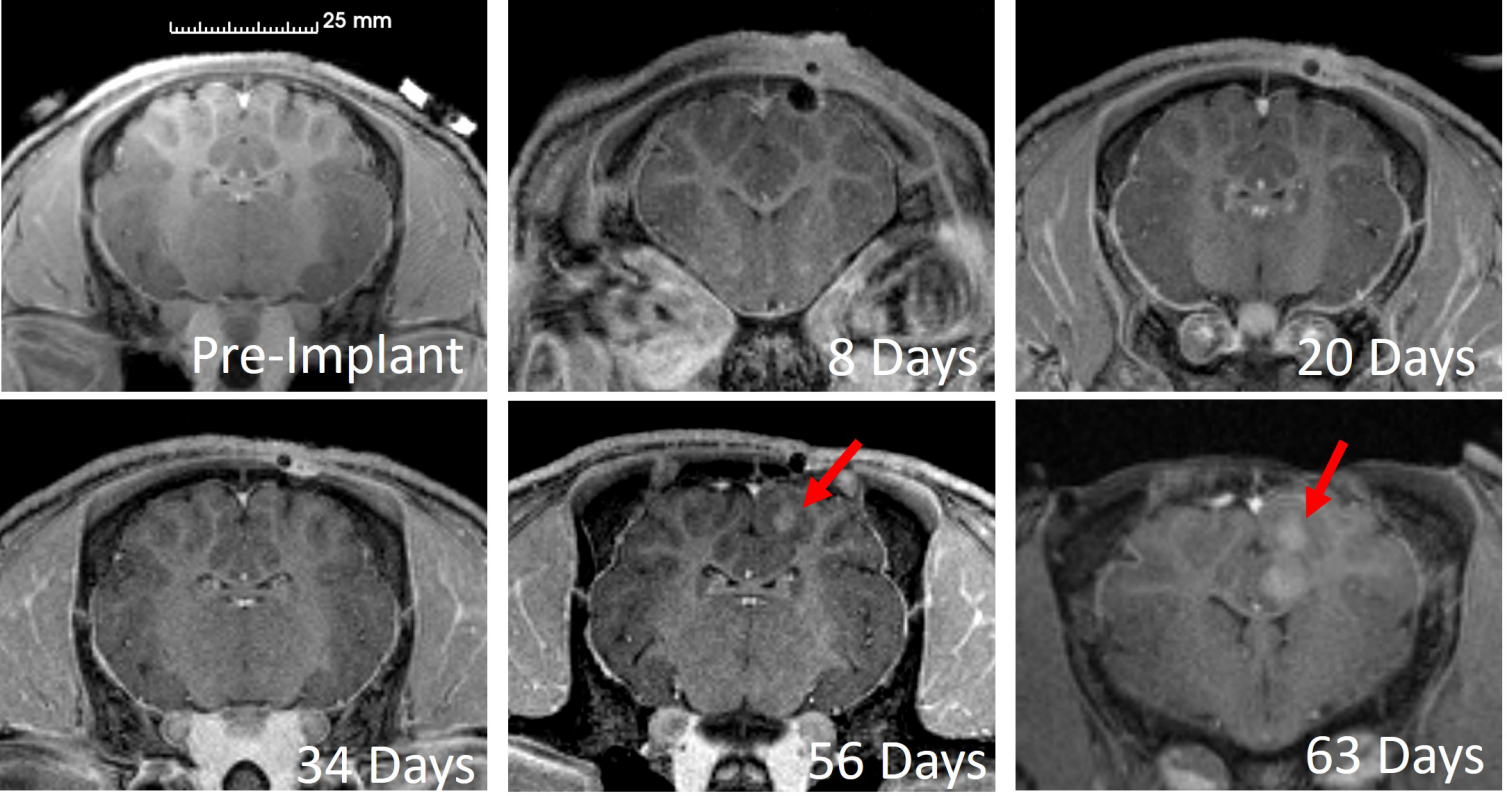
Longitudinal CE-MRI for a representative Oncopig. Tumor, depicted by the red arrow, was first observed during the imaging session 56 days after inoculation.

**Figure 2** shows selected CE-MRI images from pre-implantation through tumor growth for one animal (OP4). In this case, the burr hole from implantation is visible on the right side of each image. The first indication of tumor onset was observed 56 days after inoculation and a follow-up scan on day 63 revealed significant tumor growth between imaging sessions. Specifically, tumor volumes segmented from CE-MRI scans indicated that the tumor volume increased from 132 mm^3^ to 381 mm^3^, a factor of 2.9, over 7 days in this animal.

**Figure 3:**
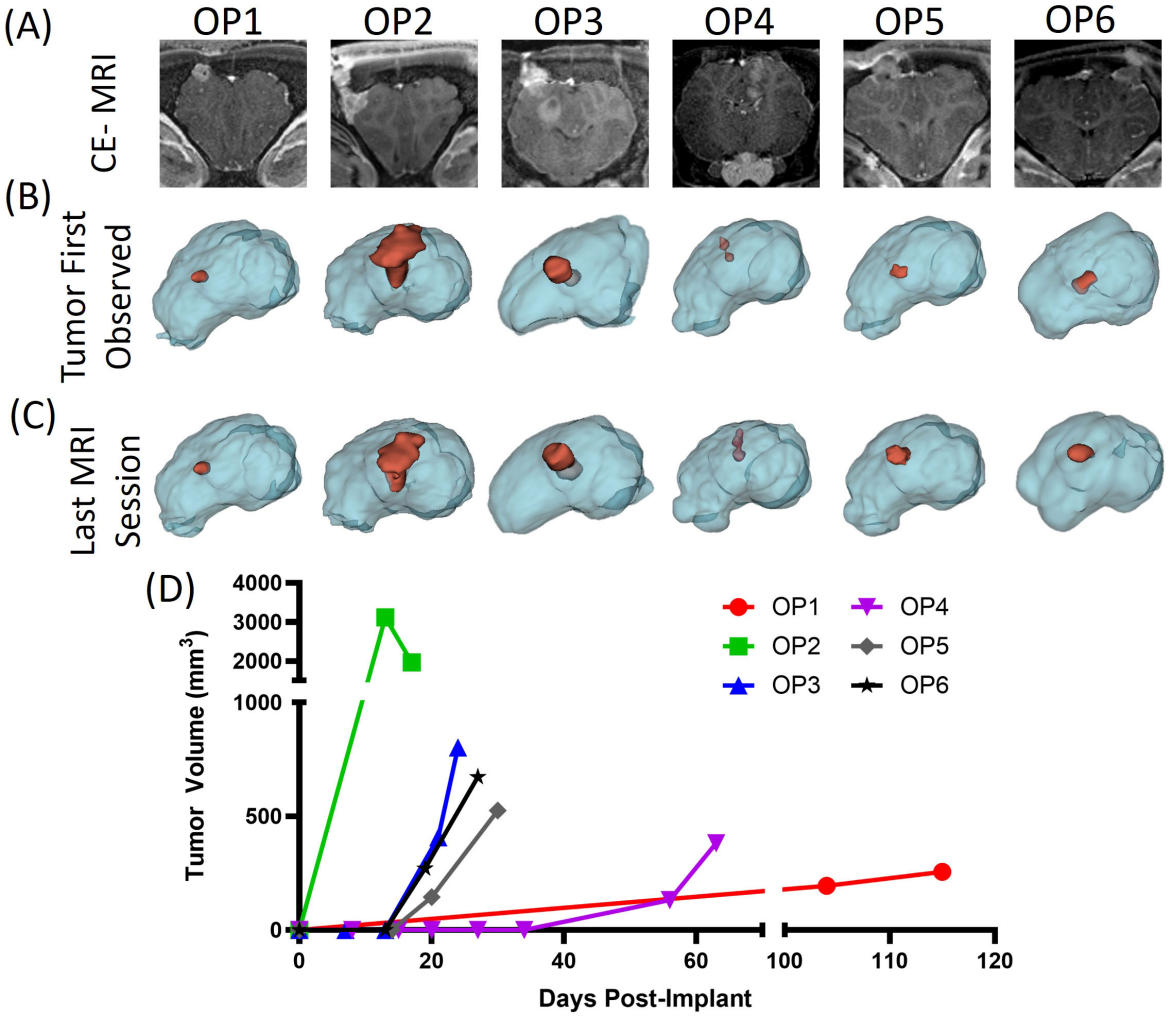
**(A)** Representative coronal T1-weighted CE-MRI slices for all pigs that exhibited tumor growth. 3-D renderings of the normal brain (blue) and tumor (red) segmented from the CE-MRI volumes in which tumor was first observed **(B)** and prior to euthanasia **(C)**. **(D)** Tumor volume (mm^3^) vs. days after inoculation for each animal.

Similar volume-based analyses were performed for all animals that exhibited tumor development on MRI and the results are provided in **Figure 3**. **Figure 3A** shows a representative coronal CE-MRI image for each animal that exhibited a tumor at the last imaging session before euthanasia. Inspection of the images reveals intersubject variability in the gross characteristics of these tumors. Some tumors extended deep into the brain and showed lobular features. In several animals, contrast enhancement indicated tumor growth that extended outside the brain and through the burr hole in the skull. Three-dimensional surface renderings of tumors and whole brain elucidate these observations and show variability in size, disbursement and morphology of the lesions **Figure 3B, C**.

**Figure 3D** provides the tumor volumes determined from segmented volumes for tumor-bearing animals, illustrating variability in tumor onset time and growth rates (Plotted using Prism version 10 (GraphPad, San Diego, CA, USA). Final volumes were 256, 1961, 803, 382, 525, and 673 mm^3^ for OP1-OP6, respectively. Except for the tumor in OP2, which decreased in size from 3116 mm^3^ to 1961 mm^3^, tumors in all animals grew between the final two imaging sessions. The tumor volume decrease in OP2 appears to be regression as the CE-MRI-defined boundaries retreated in several dimensions.

### 3.2 Histopathological analysis

**Figure 4:**
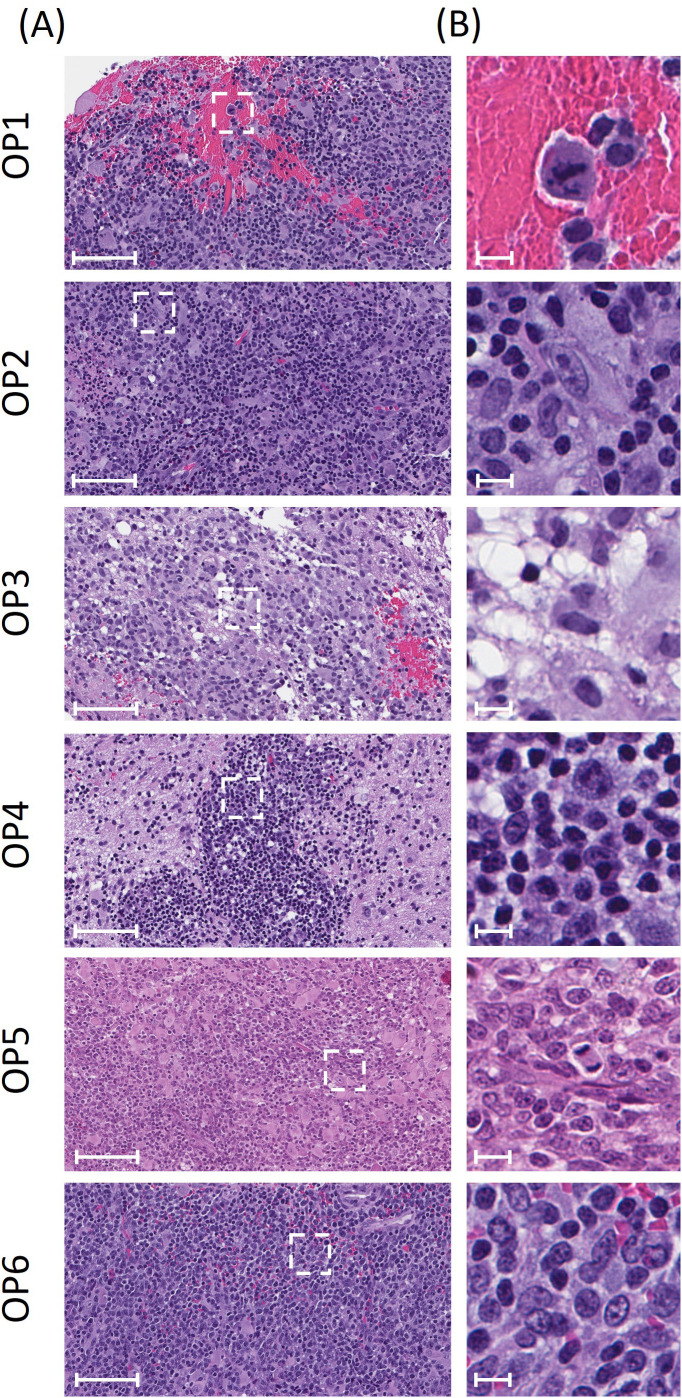
Images of H&E stained tumor specimens for OPs 1-6. Scale bars are 100 μm and 10 μm in **(A, B)**, respectively. The dotted white boxes in **(A)** correspond to the higher resolution images in **(B)**.

**Figure 5:**
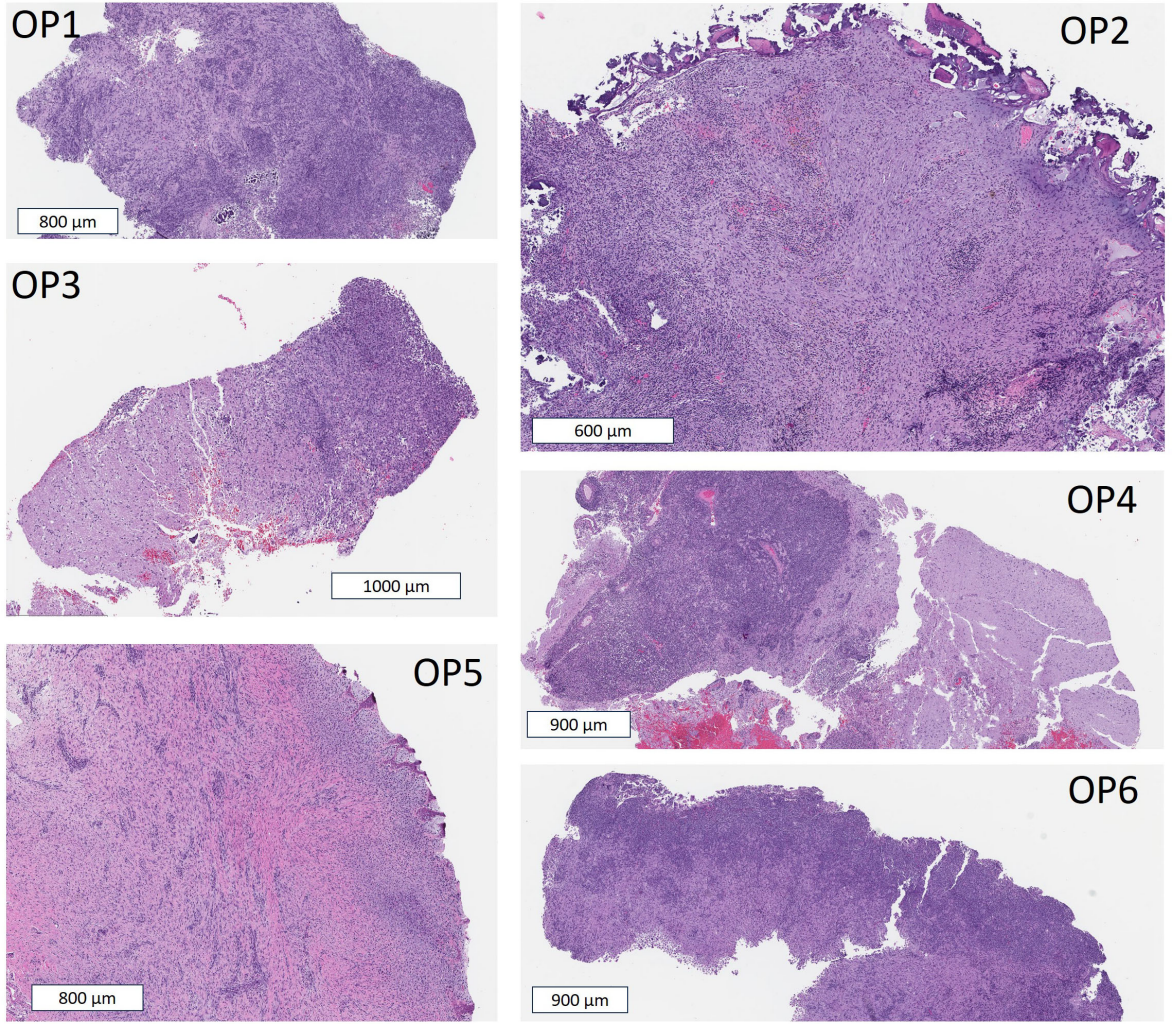
Images of H&E stained tumor specimens showing infiltrative tumor boundaries in OPs 1-6.

**Figure 4** shows representative H&E tumor specimens from each animal at two different magnifications and **Table 2** summarizes results of the pathological analysis of all slides. Tumors from all six animals showed characteristics consistent with WHO grade III high-grade glioma, including a high degree of cellularity, cellular atypia and mitotic figures. Furthermore, tissue specimens from all animals showed poorly demarcated, infiltrative tumor boundaries characteristic of diffusely infiltrating glioma (shown in **Figure 5**). Endothelial proliferation was present in specimens from OP1 (as shown in S.1) but was not observed in other specimens. None of the specimens showed necrosis.

**Table 2:** Histopathological analysis for all tumor samples per animal.

Characteristics	*OP1*	*OP2*	*OP3*	*OP4*	*OP5*	*OP6*
High-Grade	7/8	3/7	7/9	4/5	3/4	2/2
Atypia	8/8	7/7	9/9	5/5	3/4	2/2
Mitotic Figures	4/8	3/7	2/9	1/5	3/4	2/2
Endothelial Proliferation	2/8	0/7	0/9	0/5	0/4	0/2
Necrosis	0/8	0/7	0/9	0/5	0/4	0/2

Specimens stained for EGFR, OLIG2, GFAP, KRAS, and P53 are shown in S.2. Immunohistochemistry confirms that the observed tumors harbor mutations in KRAS and P53 reflecting transformation and tumor initiation using the Oncopig system. Tumors demonstrated an abundance of staining for GFAP and OLIG2, confirming that the established tumors were glial in origin. Interestingly, this finding was observed even in tumors adjacent to the skull in OP5 and OP6, indicating that they were consistent with a glial neoplasm rather than tumor formation from other tissues such as the meninges (shown in S.3). Tumors did not show EGFR overexpression based on IHC (shown in S.2).

### 3.3 Tumor resection

**Figure 6:**
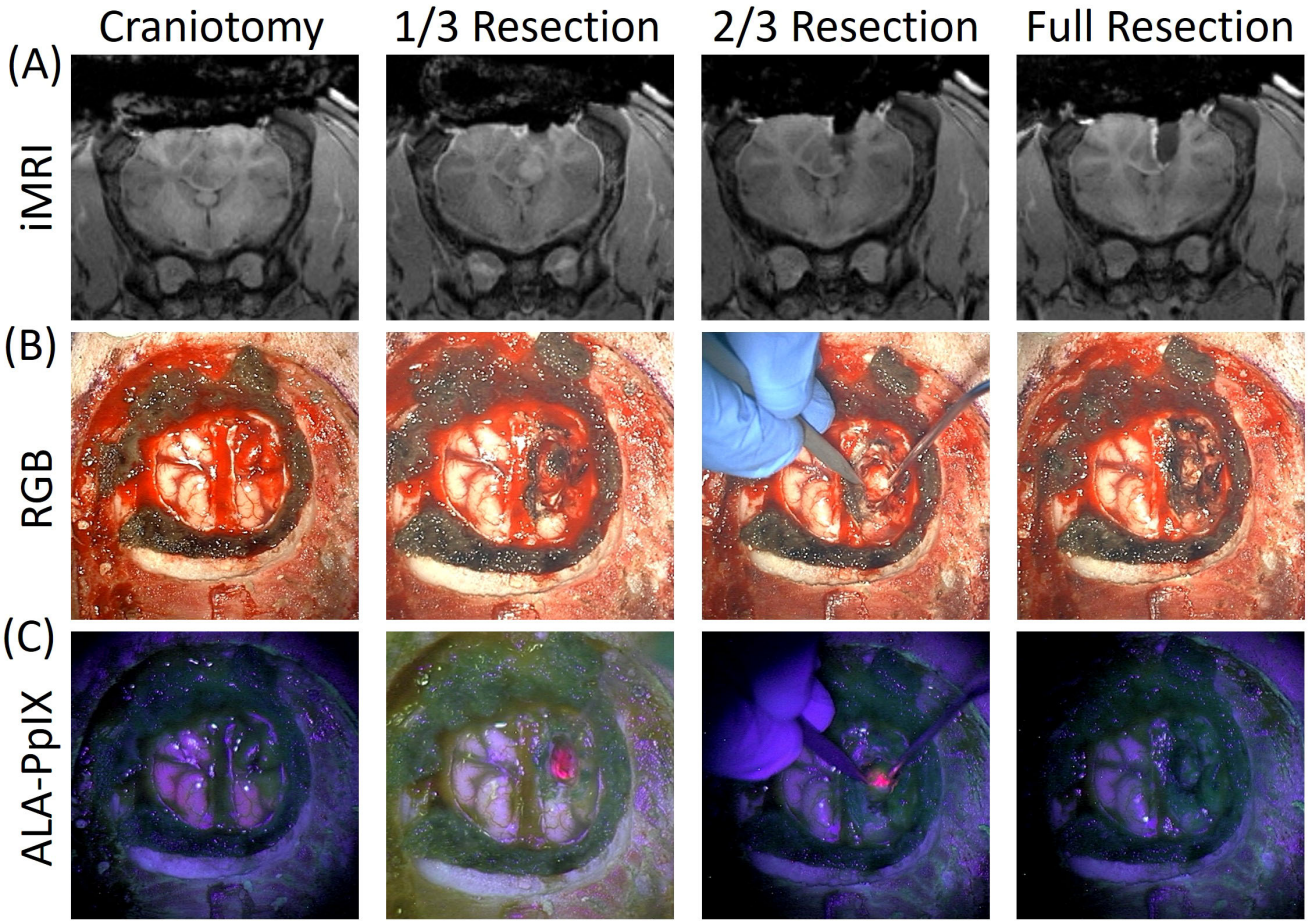
Image-guided resection of glioma in an Oncopig. **(A)** Intraoperative MRI, **(B)** widefield RGB, and **(C)** PpIX fluorescence images are displayed at multiple stages throughout the surgery. PpIX fluorescence at 1/3 and 2/3 resection was rated a 3 on fluorescence grading scale.

To explore the feasibility of using the model in a surgical setting, a single animal (OP4) underwent a craniotomy and tumor resection with 5-ALA and intraoperative MRI. Following craniotomy and tumor exposure, the lesion grossly appeared consistent with a high-grade glioma. Tumor texture was firm and visibly distinct from surrounding brain tissue under white light illumination with the operating microscope. These observations were confirmed with stereotactic image-guidance. The tumor center was not necrotic, but its margins were poorly demarcated. Additionally, under blue light illumination tumor exhibited vivid PpIX fluorescence that decreased in intensity at the margins, recapitulating clinical observations in human high-grade gliomas. **Figure 6** shows representative multimodal images (iMRI, white light, and corresponding ALA-PpIX fluorescence) acquired at different times throughout resection. The iMR images show tumor location deep in the brain and the surgical cavity as the procedure progressed. In the visible surgical field, the tumor exhibited significant enhancement with ALA-PpIX fluorescence graded visually at level 3 on a 0–3 scale, indicating a high level of PpIX concentration.

## 4 Discussion

This study reports induced glioma formation in pigs using the transgenic Oncopig model. Specifically, we showed that implanting the brains of Oncopigs with AAV’s encoding a Cre-recombinase plasmid resulted in spontaneous tumor growth in the inoculated region. To our knowledge, this study is the first to report glioma induction in this model in multiple animals.

Of the ten Oncopigs included in the study, six exhibited lesions visible on contrast-enhanced MRI which were later confirmed to be tumor. Time between inoculation and initial detection of lesions varied between animals, yet, in most pigs, tumors grew rapidly once detected (median growth rate of 36.86 mm^3^/day with a max growth rate of 131.56 mm^3^/day in OP3). The longest growth rate was observed in the first study animal (OP1) that received a single vector rather than the four-vector cocktail used for the other animals, though whether this caused the longer growth rate would need to be confirmed. Pathological evaluation of tumor specimens revealed features consistent with high grade glioma in all tumors, including diffuse borders with infiltrative features, atypia, and mitotic figures. Specimens from OP 1 tumors also exhibited endothelial proliferation; however, we did not observe this in the other tumors, nor did we observe pseudopallisading necrosis, both pathognomonic for WHO grade IV (glioblastoma). Collectively, the results suggest that the model largely recapitulates key features of human gliomas and closely correlates to WHO grade III high-grade gliomas.

The infiltrative nature of these tumors is particularly attractive for translational research involving new surgical techniques. Identifying and managing infiltrative regions around bulk tumor remain key challenges in surgical resection of human tumors. In this context, use of xenograft tumors, which usually present with well-defined borders, is less ideal for studying how novel surgical techniques perform in these regions. An infiltrative, large animal model such as reported herein appears to be well-suited to evaluate new techniques in the more challenging tumor border regions, which, to our knowledge, has not been available previously.

Options for conducting research on glioma in large animals are limited. A recent study by Hoopes et al, reported a reproducible human xenograft brain tumor model in wild type Yucatan minipigs using an immunosuppression regimen (13). This effort represented an important advance for the field, showing reliable and rapid tumor growth using well-characterized human cell lines. This model is also generally less costly than transgenic animals due to animal cost and often more rapid tumor development. However, as with many human tumor xenografts used across species, these tumors present with non-infiltrative, well-defined borders that often detach readily from normal brain tissue relative to their spontaneous tumor counterparts, making them far less suitable for studying new surgical strategies. Furthermore, effects of systemic immunosuppression alter endogenous responses to the tumors, and thus, represent additional departures from human cases. An alternative approach is enrollment of companion animals (canine and feline pets) with spontaneous tumors; however, availability and heterogeneity of disease are significant challenges for research using these subjects.

We conducted a single-subject feasibility study using ALA-PpIX fluorescence-guided surgery, which demonstrated a high level of PpIX accumulation in the tumor based on the visual scoring system used clinically. Although limited to a single subject, the ability to successfully perform this type of standard surgical resection helps demonstrate the translational usefulness of the model. Future studies of novel surgical guidance (fluorescence, navigation, intraoperative imaging, etc.), ablation, or resection techniques would benefit from evaluation of the boundary regions after tumor resection and/or tumor recurrence. Additional future efforts will include examination of intratumoral heterogeneity and additional molecular analysis, which are especially relevant for applications involving the evaluation of novel therapeutics.

The last four animals tested followed similar protocols but did not exhibit radiographic tumor on MRI, even 100 days after inoculation, a surprising result given the serial success of the first six pigs. The possible presence of anti-AAV antibodies that neutralize the activity of the viral vectors is one possible explanation. Reports from the animal supplier indicating that these antibodies are circulating throughout the colony support the hypothesis; however, further study is needed to confirm that this is the cause. If the hypothesis is correct, other less-common neurotropic viral vectors should be considered as a more productive path forward. Another potential explanation is the change from an image-guided implantation protocol to stereotactic implantation using pre-procedure CT. Although we do not believe the locations of the implantation were significantly different between the two approaches, this was not confirmed and a change in procedure cannot be ruled out. Again, further investigation is needed to identify the cause for the change in tumor take rate.

In general, imperfect tumor yields are expected across animal models and the 6/10 result reported herein is well within the range reported for tumors induced in other Oncopig studies. Previous reports have shown yields between 10 and > 90% (23, 28, 29, 33, 34). Imperfect take rates and variability in tumor growth rates can impact study costs, requiring more animals and extended housing intervals. Although we anticipate yield will improve with model refinement, the cost/benefit of using the model will need to be considered for each application. Studies for which experimental objectives can only be achieved using a large animal, infiltrative model may be well served by this system, even with the added resource requirements.

## 5 Conclusions

This study demonstrates viral-vector-based induction of brain tumors using the Oncopig platform. The resulting centimeter-scale tumors exhibited infiltrative borders and other pathological characteristics consistent with human high-grade glioma. These features indicate that this model system more closely resembles human disease than other small or large animal models reported to date; and thus, appears to be a more realistic platform for evaluating new surgical techniques and other brain tumor treatment strategies.

## Data Availability

The raw data supporting the conclusions of this article will be made available by the authors, without undue reservation.
