# Supplementary material for: Development of a high-grade glioma preclinical surgery model using an inducible KRAS/TP53 Oncopig
Source: Front Oncol. 2026 Apr 20;16:1810135. doi: 10.3389/fonc.2026.1810135 (PMC13136911; doi:10.3389/fonc.2026.1810135)
Supplement: Supplementary file 1 [file DataSheet1.docx]

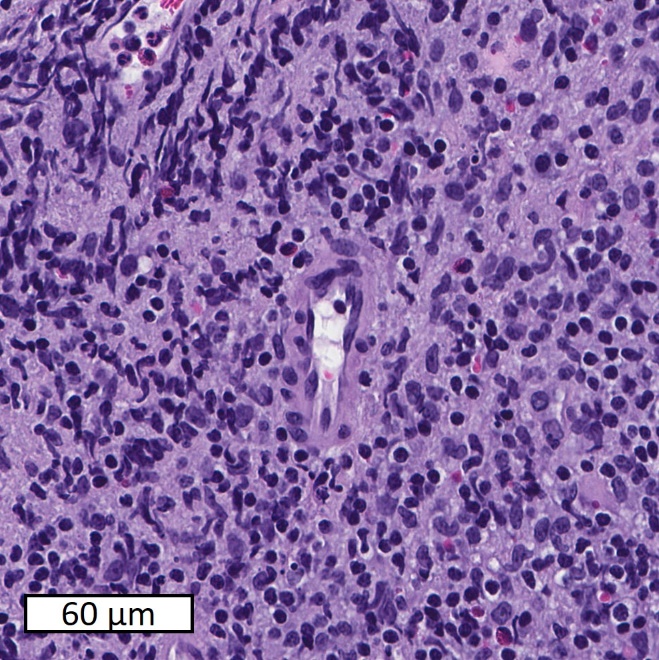


S.1. H&E slide showing endothelial proliferation in OP1


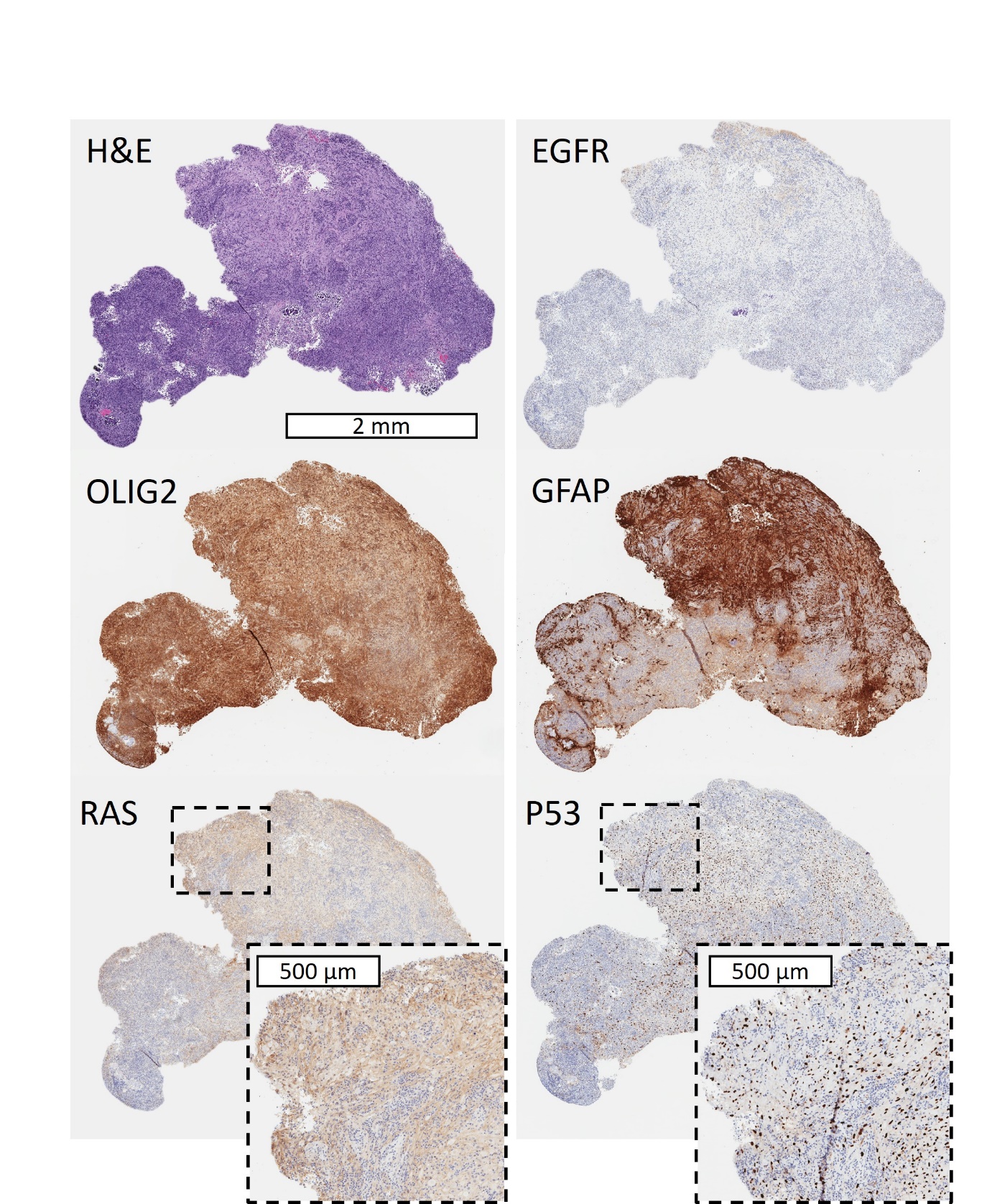


S.2. H&E, and IHC of EGFR, OLIG2, GFAP, RAS, and P53 from OP2.


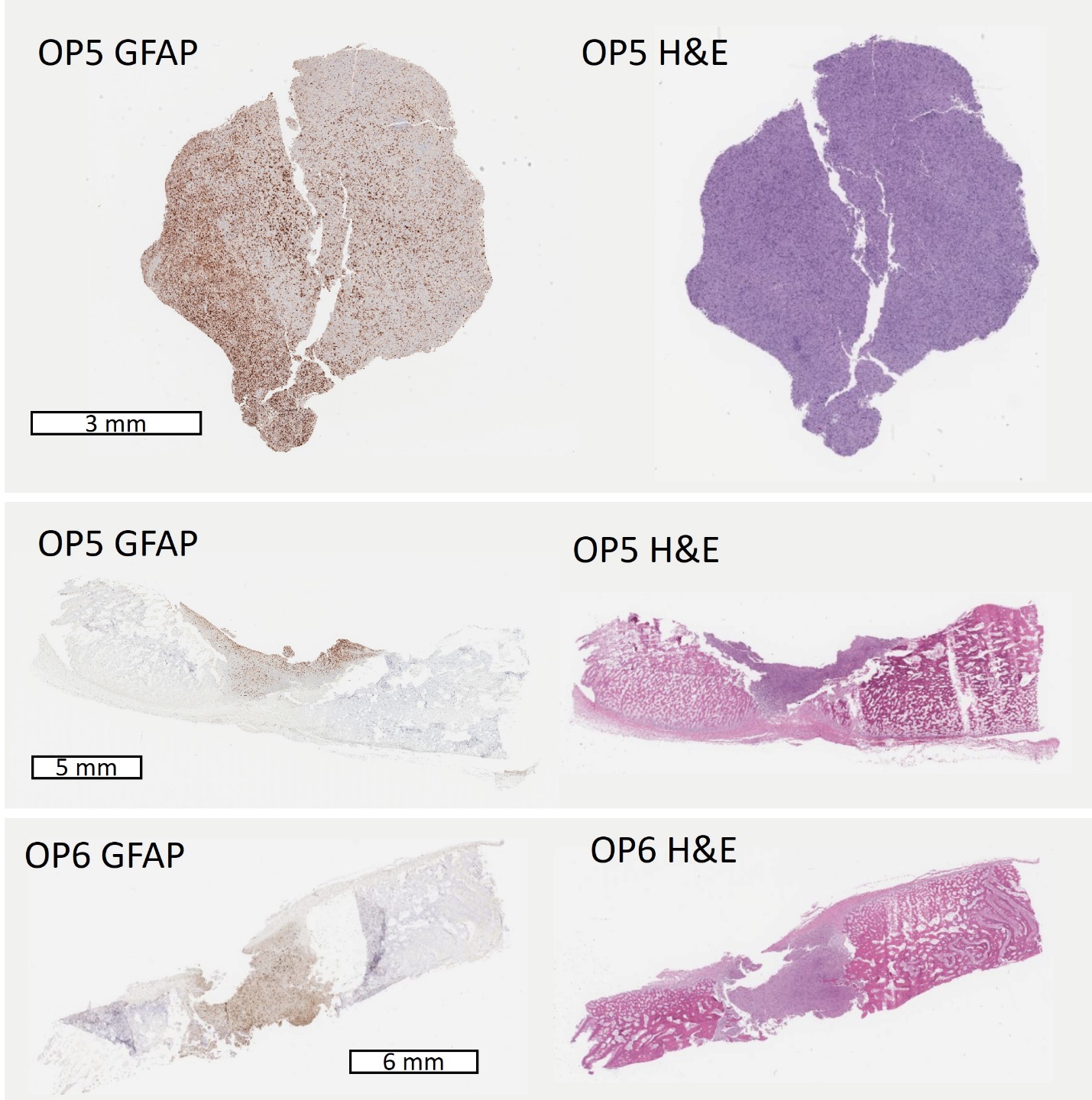


S.3. H&E and anti-GFAP IHC stained slides for OP5 and OP6 tumor samples.
