# Supplementary material for: Development of a high-grade glioma preclinical surgery model using an inducible KRAS/TP53 Oncopig
Source: Front Oncol. 2026 Apr 20;16:1810135. doi: 10.3389/fonc.2026.1810135 (PMC13136911; doi:10.3389/fonc.2026.1810135)
Supplement: Supplementary file 2 [file DataSheet2.docx]

STAINING PROTOCOL:

IHC slides were cut at 4um and air dried at room temp before baking at 60 degrees Celsius for 30 minutes. The automated protocol was performed on the Leica Bond Rx (Leica Biosystems Inc. 1700 Leider Lane, Buffalo Grove, Illinois 60089) and includes paraffin dewax, antigen retrieval and staining. Primary antibody binding was detected and visualized using the Leica Bond Polymer Refine Detection Kit (DS9800) with DAB chromogen and Hematoxylin counterstain. Positive controls were performed for each stain. Stain-specific protocols follow:

•GFAP: Heat induced epitope retrieval using Bond Epitope Retrieval 2, pH9 (Leica Biosystems, Cat# AR9640) was incubated at 100 degrees Celsius for 20 minutes. Primary antibody GFAP (Rabbit Polyclonal Antibody, Catalog# Z0334, Agilent Technologies Inc. 5301 Stevens Creek Blvd, Santa Clara, CA 95051) was applied and incubated for 15 minutes at room temperature at a 1:800 dilution.

•EGFR: Heat induced epitope retrieval using Bond Epitope Retrieval 2, pH9 (Leica Biosystems, Cat# AR9640) was incubated at 100 degrees Celsius for 20 minutes. Primary antibody EGFR (EP38Y, Rabbit Monoclonal Antibody, Catalog # ab52894, Abcam 1 Kendall Sq Ste B2304, Cambridge, MA 02139) was applied and incubated for 15 minutes at room temperature at a 1:300 dilution.

•OLIG2: Heat induced epitope retrieval using Bond Epitope Retrieval 1, pH6 (Leica Biosystems, Cat# AR9961) was incubated at 100 degrees Celsius for 20 minutes. Primary antibody OLIG2 (Rabbit Polyclonal Antibody, Catalog# PA5-85734, Thermo Fisher Invitrogen, 22 Friars Dr, Hudson, NH 03051) was applied and incubated for 15 minutes at room temperature at a 1:200 dilution.

•KRAS G12D: Heat induced epitope retrieval using Bond Epitope Retrieval 2, pH9 (Leica Biosystems, Cat# AR9640) was incubated at 100 degrees Celsius for 40 minutes. Primary antibody RAS (mutated G12D, Rabbit Polyclonal Antibody, Catalog# ab221163, Abcam 1 Kendall Sq Ste B2304, Cambridge, MA 02139) was applied and incubated for 15 minutes at room temperature at a 1:500 dilution.

•P53: Heat induced epitope retrieval using Bond Epitope Retrieval 2, pH9 (Leica Biosystems, Cat# AR9640) was incubated at 100 degrees Celsius for 30 minutes. Primary ready-to-use antibody P53 (DO-7, Ready-To-Use Mouse Monoclonal Primary Antibody, Leica Biosystems Inc. 1700 Leider Lane, Buffalo Grove, Illinois 60089) was applied and incubated for 15 minutes at room temperature.
